# Damage-Net: A program for DNA repair meta-analysis identifies a network of novel repair genes that facilitate cancer evolution

**DOI:** 10.1016/j.dnarep.2021.103158

**Published:** 2021-09

**Authors:** Aldo S. Bader, Martin Bushell

**Affiliations:** a Cancer Research UK Beatson Institute, Glasgow, G61 1BD, UK; b Institute of Cancer Sciences, University of Glasgow, Garscube Estate, Switchback Road, Glasgow, G61 1QH, UK

**Keywords:** TCGA, The Cancer Genome Atlas, ACC, adrenocortical carcinoma, DNet-genes, Damage-Net identified genes, ACC-Net genes, ACC specific Damage-Net identified genes, Mass spectrometry, Proteomics, Genomics, TCGA, Adrenocortical carcinoma, Mutational burden, Damage-Net

## Abstract

The advent of genome-wide methods for identifying novel components in biological processes including CRISPR screens and proteomic studies, has transformed the research landscape within the biological sciences. However, each study normally investigates a single aspect of a process without integration of other published datasets. Here, we present Damage-Net, a program with a curated database of published results from a broad range of studies investigating DNA repair, that facilitates simple and quick meta-analysis. Users can incorporate their own datasets for analysis, and query genes of interest in the database. Importantly, this program also allows users to examine the correlation of genes of interest with pan-cancer patient survival and mutational burden effects. Interrogating these datasets revealed a network of genes that associated with cancer progression in adrenocortical carcinoma via facilitating mutational burden, ultimately contributing substantially to adrenocortical carcinoma’s poor prognosis. Download at www.damage-net.co.uk.

## Highlights

• Damage-Net is a downloadable program for quick and easy meta-analysis of large datasets.
• Users can use the datasets in the curated database of DNA-repair based studies and also upload their own data for analysis.
• Built in pan-cancer mutation and survival analysis allows genes of interest to be queried for DNA-repair roles in cancer.
• Analysis of the database found a novel group of DNA repair genes and a major role for DNA-repair in adrenocortical carcinoma.

## 1 Introduction

A variety of DNA damaging agents assault our genomes every day, leading to the formation and subsequent repair of a range of DNA lesions. These different lesions are repaired by various repair pathways, each optimised for the resolution of a different form of DNA damage and each required for the successful maintenance of genomic integrity.

The major DNA repair pathways include base excision repair (BER), nucleotide excision repair (NER), single-strand break repair (SSBR) and double-strand break repair (DSBR) [1,2]. BER repairs non-bulky DNA adducts, such as oxidised nucleotides [3], NER repairs bulky, helix distorting DNA adducts [4], SSBR repairs single-strand cuts in the double-helix [5] and DSBR functions via multiple downstream repair pathways, predominantly non-homologous end-joining (NHEJ) and homologous recombination (HR) [6]. Despite these highly complex and efficient repair pathways, DNA repair is imperfect and can therefore lead to the formation of mutations throughout our genomes [7,8]. The accumulation of mutations can disrupt cellular functions ultimately leading to disease and is most notably the central driver of carcinogenesis [9,10]. These mutations can alter protein function, disrupting normal cellular function and, in some cases, acquiring characteristics which drive tumorigenesis. Mutagenesis can be accelerated via mutation of the genes encoding DNA repair factors, leading to impaired protein function and therefore reduced repair fidelity [11,12]. This mechanism constitutes the basis for cancer pre-disposing conditions, such as hereditary breast and ovarian cancer syndrome [11]. A broad understanding of the processes governing DNA repair is therefore critical for the development of new therapeutic strategies for cancer.

High-throughput investigations are often used to identify novel factors involved in DNA repair. These studies include proteomics of chromatin associated proteins [13,14], interactomes of known components of the DNA repair machinery [15,16] and the study of protein modifications following exposure to DNA damaging agents [17,18]. These methods have proven effective at identifying new participants in a variety of DNA repair pathways and there is therefore a wealth of published data available utilising these techniques. However, only a small fraction of the proteins identified have been investigated in depth meaning there is a substantial amount of information left unused in these datasets. In addition, the integration of multiple datasets investigating the same processes magnifies their power as successful hits can be identified with greater confidence, outlier removal is more accurate and a far greater understanding of the results can be obtained [19]. Despite these benefits, and the abundance of available data, integration of multiple published datasets or even comparisons between new data and published data are rare. We believe this is likely due to the barriers of searching for data in the literature and the bioinformatic analysis of the results.

To address this, we wrote a graphical program named Damage-Net (All downloads and information available at www.damage-net.co.uk), that simplifies the integration of large datasets, mainly proteomics and genetic screens, allowing for comparisons to be made between datasets and for commonalities to be found. Users can easily investigate the curated database of 39 published results that investigate DNA damage and upload their own results to interrogate. In addition, data from The Cancer Genome Atlas (TCGA) database has been analysed and incorporated into Damage-Net to allow users to easily determine if genes of interest correlate with mutational burden or alter survival in 33 cancer subtypes compiled by the TCGA.

In this study, we showcase the powerful capabilities of Damage-Net by using it to conduct large scale analysis on all 39 of its datasets, identifying a group of genes that are potentially novel DNA repair factors. We then conduct further analysis on these genes, finding a network of genes cooperating in adrenocortical carcinoma (ACC) that associates with mutational burden and substantially reduce patient survival.

## 2 Results

### 2.1 An overview of Damage-Net functionality and features

**Fig. 1:**
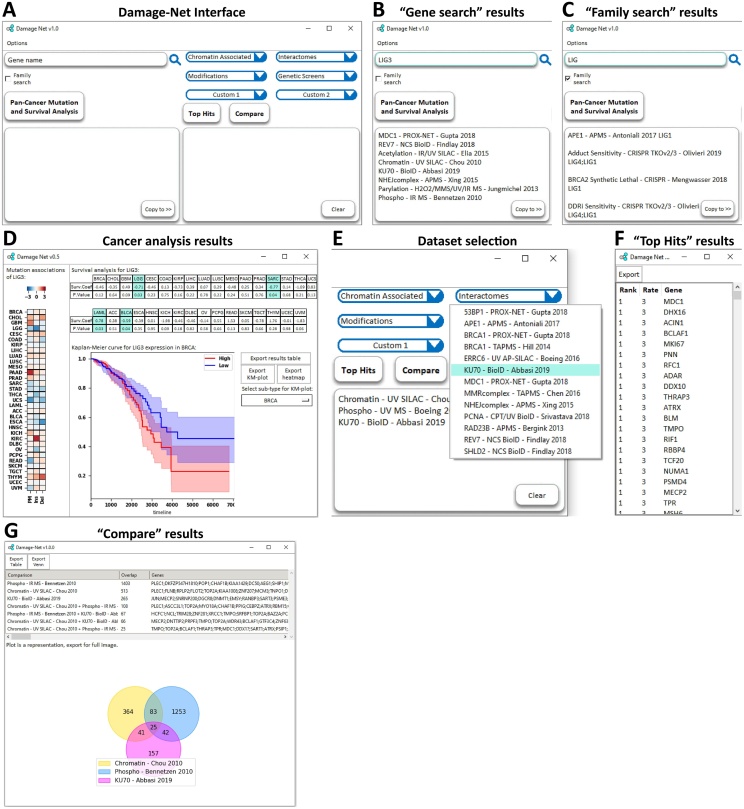
An overview of Damage-Net functionality and features. (A) Main screen of the Damage-Net interface. (B) Result window of the gene search function using Ligase 3 (*LIG3*) as an example, results are displayed in the lower box. (C) Result window of the family search function using Ligase (*LIG*) as an example, results are displayed in the lower box. (D) Result window of the Pan-Cancer Mutation and Survival Analysis function using Ligase 3 (*LIG3*) as an example, mutation association heatmap is on the left and survival analysis table and survival curve is on the right, highlighted table results are those with significant changes. (E) Example of how to select datasets for analysis with the Top Hits and Compare functions. (F) Result window for the Top Hits of the datasets selected in (E). (G) Result window for the Compare function of the datasets selected in (E).

Damage-Net was written in the Python coding language with the database backend written in SQL and was compiled to function as an independent program on Windows, Linux and Mac OS. It currently contains the results of 39 publications that investigated various aspects of DNA repair on a large-scale (Supplementary Table 1) [[13], [14], [15], [16], [17], [18], [19], [20], [21], [22], [23], [24], [25], [26], [27], [28], [29], [30], [31], [32], [33], [34], [35], [36], [37]], as well as the gene expression and mutation data for 33 cancer sub-types from the TCGA database. The proteomic datasets were all selected from peer-reviewed publications that investigated aspects of the DNA damage-response. These datasets had to be generated in a way that identified DNA-damage specific results, e.g. via comparing a DNA damage treated sample to an untreated sample or immunoprecipitation of a known repair factor. In addition, we chose to not impose selection criteria to prevent bias within the database, instead it is designed to grant easier access to currently available data. We will continue to search for and add datasets to the database, those currently available are all the datasets we have identified so far. We designed Damage-Net with simplicity and ease of use as our primary focus, to make data availability and analysis as accessible as possible. To achieve this, there are four main functions; “Gene search”, “Pan-Cancer Mutation and Survival Analysis”, “Top Hits” and “Compare” (Fig. 1A).

The “Gene search” function takes a single gene symbol, e.g. *LIG3*, and returns a list of all studies in the database that identified this gene, or its encoded protein, as a significant hit. In the case of ligase 3, encoded by *LIG3*, we find interactomes for *MDC1*, *REV7*, *KU70* and the NHEJ complex, a study on chromatin binding in response to UV and a number of modification studies (Fig. 1B). This function can instead be used to search for protein families by searching the common identifier of the gene family you wish to search, e.g. *BRCA*, *LIG*, *SF3B* etc. The result is a list of all studies that identified members of this family followed by which members were identified (Fig. 1C). For the investigation of a specific gene, the user can also conduct pan-cancer mutation and survival analysis. This function opens a results window containing a heatmap of the association of the gene’s expression with point mutations, insertions and deletions across the genomes of all cancer sub-types (Fig. 1D). It also creates a table of survival statistics for all cancer sub-types, highlighting the significant results, and generates Kaplan-Meier curves, the sub-type of which can be switched using a dropdown selection (Fig. 1D).

Both the “Top Hits” and “Compare” functions require a list of at least 2 datasets to compare which can either be selected from the dropdown lists or be copied over from the “Gene search” results (Fig. 1E). Analysing a group of datasets with “Top Hits” opens a results window containing a table of 3 columns: Rank, Rate and Gene, which correspond to the rank of the gene in the results, the number of the studies the gene was identified in and the gene symbol respectively (Fig. 1F). The “Compare” function determines the overlap in the results of all possible combinations of the datasets searched and opens a results window containing a table and a Venn diagram (Fig. 1G). The table has 3 columns: comparison, overlap and genes, corresponding to the datasets being compared, the number of genes in the overlap between the compared datasets and the genes in this overlap respectively. The Venn diagram is only computed for up to 6 studies as greater than this becomes very difficult to plot. The results of “Cancer mutation and survival analysis”, “Top Hits” and “Compare” can all be saved in multiple table formats and all plots can be saved in a variety of image formats.

Although simple in their design, these functions provide a very effective basis of analysis for the investigation of both specific targets and large datasets. No other available tools provide genomic mutation association based on gene expression, instead other tools such as cBioPortal only provide analysis of mutations within a gene of interest, not across the genome. Other tools do provide survival curve creation, such as kmplot, however at the point of this publication these tools have a more limited set of cancer sub-types. Importantly, as well as these additional features, no tool currently provides a database similar to Damage-Net allowing users to quickly and easily query published results. The integration of different datasets can be used to uncover additional aspects of the role a particular protein may play in DNA repair, for example by finding its interacting partners and by determining the modifications that are made to it. The mutation analysis made possible by the integration of TCGA data can also significantly expand on this by providing the types of mutation associated with this gene and the cancer sub-types/tissues in which this occurs. Correlating expression of a gene with the frequency of mutations across the genome is a rarely used approach for helping to define a proteins involvement in DNA repair but one of significant benefit, both inside and outside the context of cancer.

### 2.2 Damage-Net identifies a group of potentially novel DNA repair factors

**Fig. 2:**
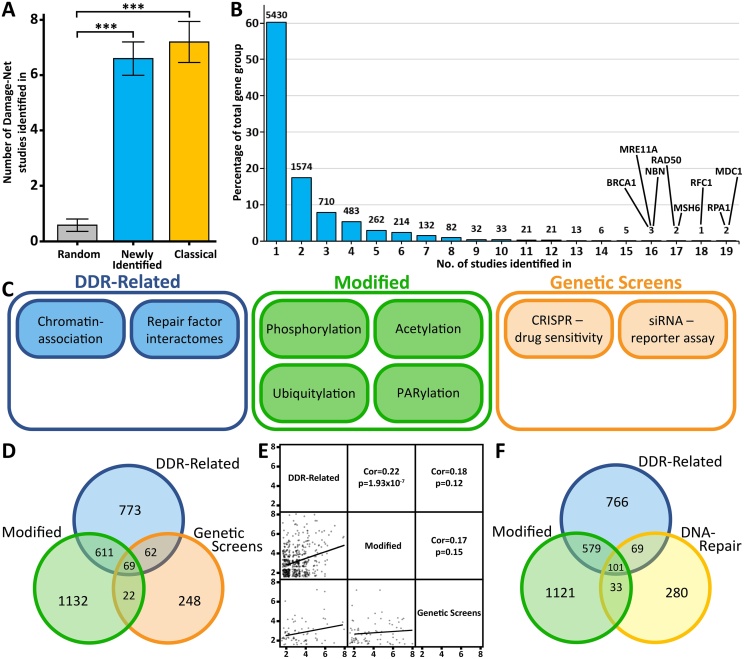
Damage-Net identifies a group of potentially novel DNA repair factors. (A) The average number of studies in which genes are identified by Damage-Net for 3 groups: randomly selected, newly identified DNA repair genes and classical DNA repair genes. Error bars represent standard error of the mean, statistical testing was done using an unpaired t-test, *** indicates p-value < 0.001. (B) Histogram of the number of datasets all genes are identified in, in the Damage-Net database, y-axis shows the percentage of total genes each group represents, number above the bars indicates the raw number of genes in each group and key results have been labelled. (C) Representation of the categorisation of Damage-Net datasets into 3 groups: DDR related, Modified and Genetic Screens. (D) Venn diagram of the genes occurring more than once in each of the three groups from (C). (E) Correlation matrix between the three groups from (C) of the number of studies in each group each gene is identified in, correlation testing was done with the Spearman Rank model. (F) Venn diagram of the genes in the DNA-repair gene ontology group, the DDR-Related gene group and the Modified gene group.

Initially we tested the capability of Damage-Net to selectively identify DNA repair factors. To do this we took 3 groups of genes; a group of classical DNA repair factors, a group of randomly selected genes and a group of DNA repair genes identified since 2013 to represent newly discovered DNA repair genes that would be the target of these investigations. Each group consisted of 40 genes which were searched using “Gene Search” for the number of datasets that identified them and the average number of datasets were then compared (Fig. 2A). This showed that both classical DNA repair and newly discovered DNA repair genes are similarly likely to be identified in ∼7 studies, whereas random genes on average were identified in <1 study. This suggests that the gene search function is selective for DNA repair factors while unrelated genes are not enriched.

Comparing the results of multiple published studies also has significant advantages for data analysis. By combining studies, we can reduce off-target hits that are often specific to a particular methodology, while true hits can be enriched [17,20]. Multiple DNA-repair studies have previously conducted and combined several proteomic approaches in order to elucidate results shared between the approaches [17,20,25]. Target genes by these approaches tend to be more accurate as they have been consistently identified across multiple approaches [[38], [39], [40], [41], [42]]. In addition, the combination of different methodologies can provide additional information as to the processes these genes may be involved in with different experimental phenotypic outputs, resulting in more in-depth conclusions. For example, 53BP1 and BRCA1 have an antagonistic relationship to promote either non-homologous end-joining (NHEJ) or homologous recombination (HR) repair of DSBs respectively. However, interactomes of both 53BP1 and BRCA1 identify each other as well as the 53BP1 co-operator RIF1 and the BRCA1 co-operator BARD1. From this, it is not possible to determine the relationship between these proteins or their interactors, however by comparing these results with that of a genetic screen investigating positive regulators of HR, only BRCA1 and BARD1 are identified as common between all three studies (Supplementary Fig. 1A). This indicates that BRCA1 and BARD1 interact to promote HR and although BRCA1 interacts with 53BP1 and RIF1 they are dispensable for the process of HR. Therefore, by combining datasets of differing methodologies, a more in depth understanding of the results can be obtained. Interestingly, the other genes identified in all three of the studies in this example included the HR factors BRCA2 and RPA2 alongside HMBOX1 and ZNF207, both of which have recently been showed to have roles associated with the DNA damage response [[43], [44], [45], [46]].

To demonstrate the capabilities of Damage-Net, we chose to integrate all 39 datasets together to identify a high-confidence group of novel DNA repair factors. Initially, we ranked all genes by the number of studies they were identified in to find the most enriched genes across all datasets. This found that the genes with the strongest enrichment in these studies were a variety of well known DNA repair factors, such as *BRCA1*, *MSH6* and the full MRN complex (*MRE11*, *RAD50*, *NBN*) (Fig. 2B). We believe that in conjunction with our previous investigation of the gene search function, this suggests that Damage-Net is effective at selectively identifying DNA repair genes and that DNA repair genes are enriched within the database. However, we also noticed that whereas over 9000 genes were identified in total, over 60 % of these were identified in only one of the 39 studies investigating DNA repair (Fig. 2B). There are likely a number of contributors to this, though since Damage-Net contains such a wide variety of datasets, a wide range of results is to be expected. To further analyse this phenomenon, we paired datasets that used comparable approaches to see if this variation was due to the inherent differences between the designs of our 39 studies. Comparing two studies that used Stable Isotope Labeling by/with Amino acids in Cell culture (SILAC) to identify proteins binding to chromatin in response to UV irradiation found only a 5–8 % overlap and comparing two BRCA1 interactome studies found only a 13–21 % overlap (Supplementary Fig. 1B). Pairing studies that investigated proteins that are either phosphorylated, ubiquitylated or acetylated in response to DNA damage fared slightly better, though still only had overlaps of 15–38 % (Supplementary Fig. 1C). A pair of genetic screens showed the lowest overlap of only 1–2 % (Supplementary Fig. 1D), however this is likely due to the different targets screened by these two studies since both derived their targets through separate processes. These results suggest that differing experimental approaches partly contribute to the results of Fig. 2B, though there may also be an element of random variation from false-positive results since the paired studies also showed significant variability (Supplemental Fig. 1B-D). However, it should be noted that even these paired studies, although examining similar processes, can have a number of experimental differences such as cell lines, treatment types and doses, incubation periods and sample preparation. This further supports our hypothesis for integrating multiple datasets, as by comparing analogous studies we can isolate the high-confidence, commonly identified results and exclude those that are cell-type or method specific.

Therefore to extract a high confidence group of hits from these datasets, we categorised each study into three groups; DNA-damage response (DDR)-related, which comprises chromatin association and repair factor interactome studies, modified, which comprises all damage induced modification studies, and genetic screens, which includes genome-wide CRISPR sensitivity screens and RNAi based reporter assay screens (Fig. 2C). To remove the large number of low confidence hits, each group of genes was characterised as the genes identified in at least 2 studies of the groups from Fig. 2C.

Comparing these gene groups found that there is a significant overlap between the DDR-related and modified groups, but minimal overlap with the genetic screens group (Fig. 2D). In addition, correlation analysis of the frequency each gene is identified in these groups found a significant correlation between the DDR-related and modified groups, but not between either of these groups and the genetic screens group (Fig. 2E). Due to these low correlations and the lack of consistent results with this group and the others we decided not to include genetic screens in the analysis, as they limit our ability to combine datasets. However, it should be noted that this reduced overlap with the other groups is likely due to the significant experimental differences between them and that these genetic screens do produce accurate results. For example, comparing the group of genetic screen results with the DNA repair gene ontology gene group found that ∼26 % of the genetic screen results were known DNA repair genes (Supplementary Fig. 1E).

We then took the comparison of the DDR-related and the modified groups and to only focus on the genes that are novel repair genes, we compared these with the DNA-repair gene ontology group (Fig. 2F). We found a reassuring overlap of 101 genes between the 680 Damage-Net identified genes and the 483 known DNA repair genes. We therefore carried forward these 579 genes in the overlap between the DDR-related and modified groups for further analysis. For simplicity, we termed this group of Damage-Net identified genes “DNet-genes”.

### 2.3 DNA repair factors and DNet-genes are associated with high mutational burden and poor-prognosis in cancer

**Fig. 3:**
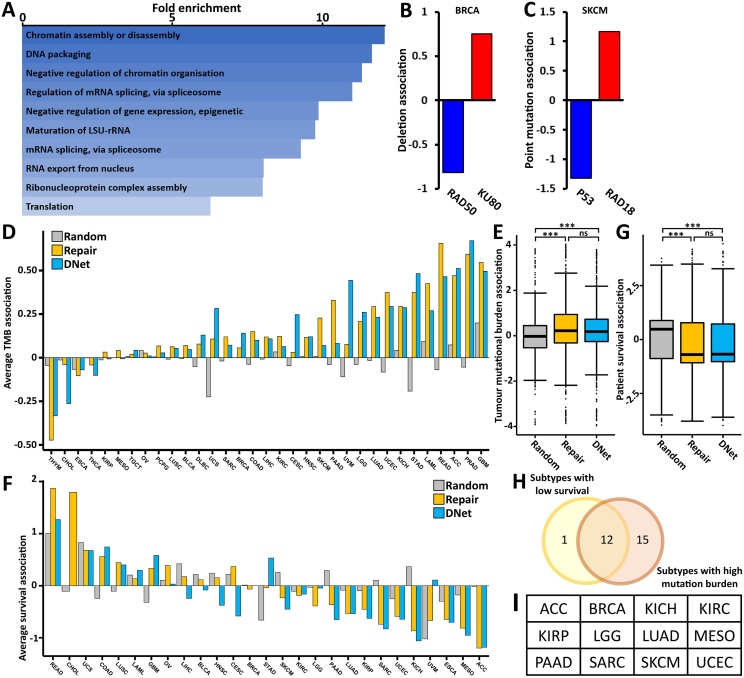
DNA repair factors and DNet-genes are associated with pan-cancer high mutational burden and poor-prognosis. (A) Gene ontology slim biological process enrichment of the 579 DNet-genes. (B) Deletion association of RAD50 and KU80 with deletions in breast invasive carcinoma. (C) Point mutation association of P53 and RAD18 with point mutations in skin cutaneous melanoma. (D) Average mutational association of three gene groups: randomly selected genes, canonical DNA repair genes and Damage-Net identified genes across all cancer sub-types. (E) Boxplot of pan-cancer tumour mutational burden association of all genes in 3 groups; random selected, canonical DNA repair genes and DNet-genes. (F) Average survival association of the genes in our three gene groups across all cancer sub-types. (G) boxplot of pan cancer survival association of all genes in our three gene groups. (H) Comparison of cancer sub-types in which DNA-repair genes are associated with low survival to those in which they are associated with increased mutational burden. (I) Table of the 12 cancer sub-types: ACC, BRCA, KICH, KIRC, KIRP, LGG, LUAD, MESO, PAAD, SARC, SKCM and UCEC, in the cross-section of (G). Statistical testing was done using an unpaired, directional Wilcox test with Bonferroni correction, ns indicates p-value > 0.05, *** indicates p-value < 0.001.

We first conducted gene ontology enrichment to understand the composition of the 579 DNet-Genes. This found a significant enrichment for genes related to various chromatin organisation and RNA-related processes (Fig. 3A). This suggests that the genes may be more peripheral to the repair process, facilitating chromatin remodelling around damage sites rather than specifically contributing to DNA processing.

To investigate possible DNA repair roles for these DNet-genes in cancer we conducted a mutation association study. To do this for a gene we first split patients into two groups; low and high expression for the gene of interest, then calculate the log2 fold change of the average mutation frequency in the high/low expression group. This allows us to determine how expression of a gene correlates with the mutational burden in a given cancer sub-type and can be very mechanistically informative. For example, breast cancer is commonly deficient in homologous recombination (HR) resulting in increased mutations due to repair of double-strand breaks via the more error-prone non-homologous end joining (NHEJ) [47,48]. When we look at the association of the HR factor *RAD50* with deletions in breast cancer, we find a strong negative association indicating that loss of *RAD50* promotes mutations whereas the NHEJ factor *KU80* shows a strong positive association indicating higher *KU80* expression promotes mutation (Fig. 3B). This analysis is a powerful tool in investigating DNA repair in cancer as we can also characterise tissue and mutation specific mechanisms of repair factors, e.g. in contrast to our previous example, the UV-response gene *RAD18* associates positively with point mutations in skin cutaneous melanoma whereas *P53* is negatively associated (Fig. 3C).

We calculated the average association of all DNet-genes with tumour mutational burden in all cancer sub-types and compared this to the averages for canonical DNA-repair genes and a randomly selected group of genes (Fig. 3D). This found that both the canonical DNA repair and DNet-gene groups were associated with increased tumour mutational burden in most cancer sub-types, whereas random genes showed few significant changes. To get an overall view on the effect these genes are having on tumour mutational burden, we compared the effect of each gene on mutational burden in each of our 3 gene groups (Fig. 3E). Compared to random genes, both DNA repair and DNet-genes show a significant increase in tumour mutational burden association. Interestingly, comparing DNet-genes to DNA repair genes shows no statistical difference, suggesting the DNet-genes cause a comparable increase in tumour mutational burden to canonical DNA repair genes. An association between DNA-repair gene activity and altered tumour mutational burden is well documented [49,50], though interestingly this is commonly identified as reduced activity increasing mutations, unlike our observation here.

Next, we used a similar analysis to investigate the effect of DNet-genes on patient survival. Comparing the average survival change for each group across the different cancer sub-types found that there is significant variation between the sub-types, but that DNA-repair genes and DNet-genes are more commonly associated with an average decrease in survival (Fig. 3F). An overall analysis, similar to that done previously, found that both DNA repair genes and DNet-genes generally show a significant decrease in survival whereas the random control group shows a subtle skew to increased survival (Fig. 3G). Again, we found no statistical difference between the repair and DNet-genes, suggesting that these groups have very similar effects on both mutational burden and patient survival. All mutation and survival associations for all gene groups are available Supplementary Table 2.

Interestingly, comparing the sub-types where DNA-repair genes cause increased mutational burden with those in which they cause decreased survival found a large overlap between the two groups (Fig. 3H-I). This indicates that these two features may be linked, such that the increased mutational burden drives the decreased survival.

### 2.4 A sub-group of DNet-genes lower cancer prognosis by increasing mutational burden

**Fig. 4:**
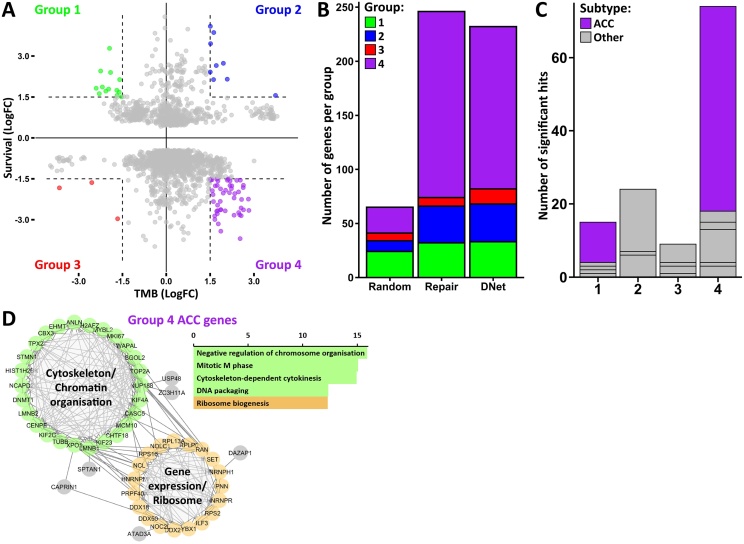
A sub-group of DNet-genes lower cancer prognosis by increasing mutational burden. (A) Association with tumour mutational burden (TMB) vs association with survival of all DNet-genes in all cancer sub-types with the 4 groups of cooperative change labelled groups 1-4. (B) Size comparison of the groups from (A) for DNet-genes against those for canonical DNA repair genes and randomly selected genes. (C) Comparison of group sizes from (A) split by ACC and all other cancer sub-types. (D) Protein interaction network (left) and gene ontology slim biological process enrichment (right) for all group 4 genes from (A) that are in adrenocortical carcinoma (ACC).

To investigate this link, we used an integrated analysis of all DNet-genes in all cancer sub-types for their effect on mutational burden and survival simultaneously. This allowed us to characterise 4 groups of coupled change: group 1 where genes associate with reduced mutations and increased survival, group 2 where genes associate with increased mutations and increased survival, group 3 where genes associate with decreased mutations and decreased survival and group 4 where genes associate with increased mutations and decreased survival (Fig. 4A). The DNet-genes show a distinct shift into group 4, demonstrating that the increase in tumour mutational burden and the decrease in patient survival shown previously are directly linked. The survival curves for a group 4 example, *KIF23* in adrenocortical carcinoma, is shown in Supplementary Fig. 2C and for a group 2 example, *USP34* in rectal adenocarcinoma, is shown in Supplementary Fig. 2D. When conducting this analysis on randomly selected genes, it showed very few hits in all 4 groups (Supplementary Fig. 2A), whereas the DNA repair genes showed a very significant shift into group 4 (Supplementary Fig. 2B). This follows our previous results that found DNet-genes and DNA repair genes to have comparable effects of significantly increased mutation association and significantly decreased survival association (Fig. 3E, G). This result therefore suggests that both DNA repair and DNet-gene expression are commonly associated with increased tumour mutational burden that drives a decrease in patient survival in cancer. Additionally, the strong similarity between the effects of DNA repair genes and DNet-genes support the hypothesis that DNet-genes are previously uncharacterised DNA repair genes, either directly involved in DNA processing or are factors associated with the repair process.

Calculating the size of each of the 4 groups from Fig. 4A showed that compared to random genes, DNA repair and DNet-genes have a substantial enrichment for group 4 (Fig. 4B). A notable feature of the DNet-gene hits in group 4 is that they appear to be dominated by hits in adrenocortical carcinoma (ACC) (Fig. 4C) which was also found to be one of the most significantly changing sub-types with DNet-genes for both mutational burden and survival (Fig. 3D, F). This means that there are large groups of DNet-genes whose expression collectively correlates with both mutational burden and decreased survival in specific cancer sub-types. We therefore conducted functional analysis on the group 4 ACC genes using gene-ontology enrichment and network analysis to determine their biological roles. The group 4 ACC genes primarily consist of genes relating to cytoskeleton/chromatin organisation and gene expression/ribosomes (Fig. 4D) which we found particularly interesting in light of the burgeoning fields of RNA binding proteins and chromatin remodellers in DNA repair [[51], [52], [53], [54], [55], [56], [57]].

The group 4 enrichment of canonical DNA repair genes (Supplementary Fig. 2B) suggests that it is common for these genes to associate with high mutational burden that drives decreases in survival. Given that the group 4 DNet-genes seem to almost entirely consist of an individual cancer sub-type and a specific set of genes, we chose to investigate this gene network further.

### 2.5 Network analysis in ACC discovers DNet-genes are part of a larger co-expression network

**Fig. 5:**
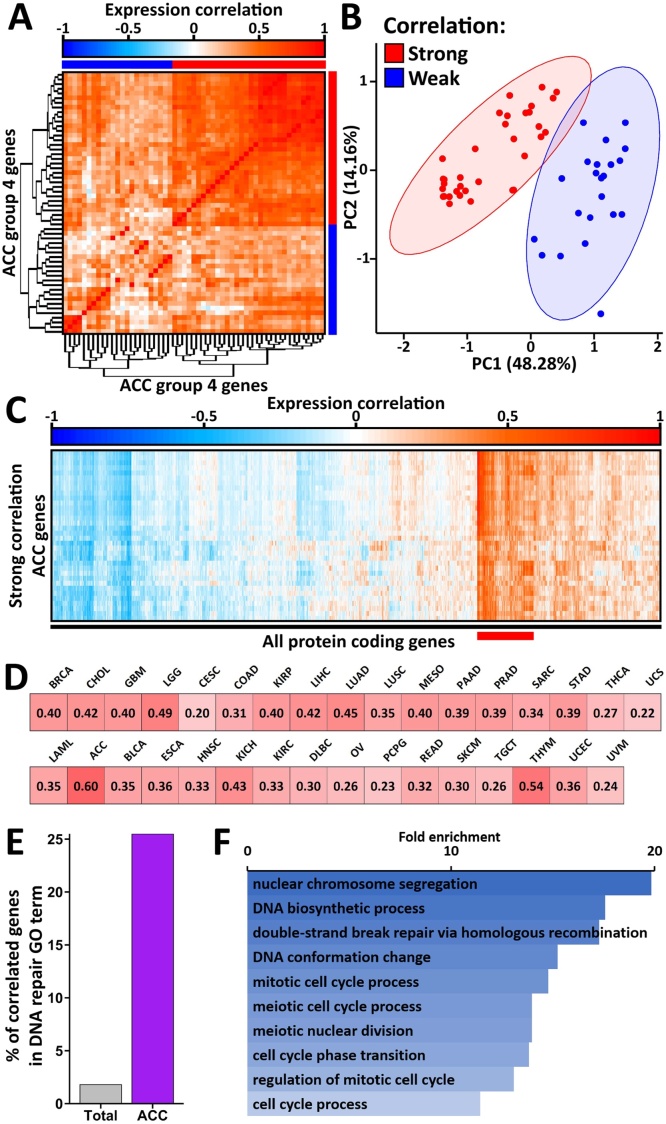
Network analysis in ACC discovers DNet-genes are part of a larger co-expression network. (A) Heatmap of expression correlation between all group 4 ACC DNet-genes with 2 groups marked with coloured bars: strong correlation (red) and weak correlation (blue). (B) Principal component analysis of the correlation matrix from (A) with the correlation groups coloured. (C) Heatmap of expression correlation between the strong correlating genes from (A-B) (y-axis) and the entire protein-coding transcriptome (x-axis) with a strong correlating group marked with a red bar. (D) Average expression correlation between all the strong correlating genes extracted from (C) in all cancer sub-types with a red colour scale applied. (E) Bar plot of percentage of strong correlating genes extracted from (C) that are DNA repair genes compared to the percentage of the total transcriptome. (F) Gene ontology slim biological process enrichment of the strong correlating genes extracted from (C).

Since the group 4 ACC genes all show similar results in our analysis, we used hierarchical clustering on their expression in ACC to determine their co-expression (Fig. 5A). This found that over half of these genes strongly co-express in ACC, which we were able to validate via principal component analysis (Fig. 5B). The tight co-expression of these genes suggests they form a network in ACC that could encompass genes beyond the original set identified by Damage-Net. Since we originally identified these genes through proteomic investigations, we may have only identified a small fraction of this ACC network due to the identification biases of these proteomic approaches [[38], [39], [40], [41], [42],^58^]. It is therefore possible that this network is much larger and consists of a significant number of genes that are elusive to proteomic investigations.

To address this, we used the same hierarchical clustering approach as before to assess the co-expression of our genes with the entire protein coding transcriptome (Fig. 5C). This indeed identified a surprising number of genes that strongly co-express with our correlating group 4 ACC genes. We filtered this matrix for the strongest correlations to gain a group of 204 genes which, combined with the 34 genes with strong correlations from the group 4 ACC genes, gave us 238 genes in this network, termed ACC-Net. It is possible that this network collectively coordinates the high mutational burden and low survival that we observed in ACC. To determine if this co-expression network is only a feature of ACC or is a more general biological feature, we calculated the average co-expression between ACC-Net genes in all cancer sub-types (Fig. 5D). This showed that these genes co-express to varying levels in all sub-types, but that their co-expression is strongest in ACC, suggesting they are part of a consistent biological pathway that is of particular importance in ACC.

To gain a general understanding of the representation of DNA repair genes in the ACC-Net group, we calculated the percentage of ACC-Net genes that are in the DNA repair gene ontology group (Fig. 5E). Over 25 % of the ACC-Net genes were found to be known DNA repair genes compared to <2% of the total transcriptome. Gene ontology of the 238 genes found that most genes were associated with cell cycle regulation with some associated with DNA conformation/biosynthesis and a substantial enrichment for DNA double-strand break repair (Fig. 5F). The large enrichment and variety of cell-cycle related processes was particularly striking and so a more in-depth gene ontology investigation was conducted to further understand this. This found that the cell-cycle related genes were primarily associated with the processes of DNA replication, chromatin organisation and cytoskeletal/mitotic spindle regulation, rather than regulation of cell-cycle progression or checkpoint regulation (Supplementary Fig. 3A-B). This gives us valuable insight into the possible functions this network may be facilitating, suggesting they may be a part of DNA repair processes specifically regarding DNA replication and via regulating chromosome segregation.

### 2.6 The ACC-Net gene network strongly associates with mutational burden and decreased survival in ACC

**Fig. 6:**
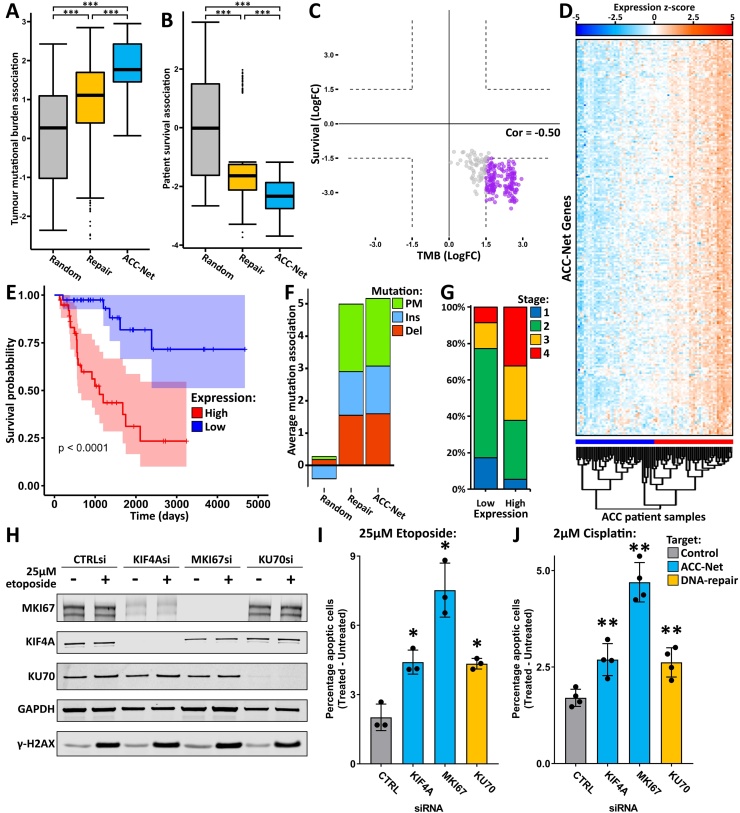
The ACC-Net gene network strongly associates with mutational burden and decreased survival in ACC. (A) Boxplot of tumour mutational burden association in ACC of all genes in 3 groups; random selected, canonical DNA repair genes and ACC-Net genes. (B) Boxplot of ACC survival association of all genes in our three gene groups. Statistical testing was done using an unpaired, directional Wilcox test with Bonferroni correction, *** indicates p-value < 0.001. (C) Association with tumour mutational burden (TMB) vs association with survival of all ACC-Net genes in ACC, Pearson correlation coefficient is marked. (D) Heatmap of ACC-Net gene expression in all ACC patient samples clustered into groups of cumulative high (red) and low (blue) expression. (E) ACC survival curve of high vs low cumulative expression of all ACC-Net genes. (F) Average mutation association of our three gene groups for point mutations, insertions and deletions separately. (G) Comparison of ACC cancer stage distribution for patients with high or low cumulative expression of all ACC-Net genes. (H) Western blot of ACC-Net target knockdowns and KU70 positive control knockdown treated with either mock or 25μM etoposide treatment. (H) Annexin V apoptosis assay of BJ-5ta cells treated with 25μM etoposide in different siRNA mediated knockdowns. Y-axis is the percentage of cells that were apoptotic in the treated minus the apoptotic percentage of the untreated sample. (J) Same as (I), but for 2μM cisplatin treatment.

To assess the effect of the ACC-Net gene expression on ACC we employed a similar analysis to that used for the DNet-genes to investigate mutational burden and survival. Comparing the mutational burden association of these genes with that of all DNA repair genes and our randomly selected control group we found that in ACC, DNA repair genes cause a significant increase in mutational burden, but that the ACC-Net genes cause an even greater increase (Fig. 6A). In fact, the expression of every gene in this group correlated with an increase in mutational burden. Conducting a similar comparison to assess changes in ACC patient survival showed that every ACC-Net gene is associated with significantly decreased survival and overall, they show an even greater decrease in survival than DNA repair genes (Fig. 6B). This unsurprisingly results in all ACC-Net genes being simultaneously associated with increased mutational burden and reduced survival; however, their association appears to be more continuous than we have seen previously as there is a significant negative correlation between mutational burden and survival (Fig. 6C). In comparison, the results from canonical DNA repair genes showed more variable results and our randomly selected control genes only had a few very variable hits (Supplementary Fig. 4A-B). Expanding our analysis to all cancer sub-types found that ACC-Net genes still showed a strong simultaneous association with increased mutational burden and decreased survival (Supplementary Fig. 4C), and was far greater than that found for DNet-genes (Fig. 4A) or DNA-repair genes (Supplementary Fig. 3B). Combined with our previous finding that these genes co-express in all sub-types (Fig. 5D), this suggests that although high expression of this gene network is most significant in ACC, that it still has an important role in general and also supports the accuracy of our findings in ACC. ACC-Net mutation and survival associations are available in Supplementary Table 2.

These results are indicative of the individual effect of ACC-Net genes; however, since we believe these genes to be operating in conjunction with each other, we needed to assess the impact of their collective expression. To determine the cumulative effect of ACC-Net genes, we grouped ACC patients into high and low expression groups based on the cumulative expression of all these genes (Fig. 6D). These groups allow us to assess the overall effect expression of the whole network has on ACC. High expression of the network results in a highly significant drop in survival, reducing the 5-year survival rate from ∼80 % in low expression patients to ∼30 % in high expression patients (Fig. 6E). This is a greater decrease than compared to the cumulative effect of DNA repair gene expression (Supplementary Fig. 4D) and overall expression of our random control genes showed no change in survival rate (Supplementary Fig. 4E). In addition, overall ACC-Net expression strongly associates with increased rates of point mutations, insertions and deletions (Fig. 6F), an increase that was slightly greater than the overall effect of all DNA repair genes. As a final piece of analysis, we asked the question of what effect ACC-Net gene expression has on tumour stage. High expression of ACC-Net genes strongly skews towards late stage, metastatic ACC, whereas low expression skews towards early-stage ACC (Fig. 6G). This indicates that ACC-Net gene expression not only reduces survival, but also advances cancer progression to metastasis.

We then tested these findings by replicating this analysis using an alternative dataset that is independent of the TCGA [59]. This dataset includes gene expression quantification via microarray, mutation quantification via exome sequencing and patient outcome data. We found strikingly similar results with this dataset as with the TCGA data (Supplemental Fig. 4F–H); increased expression of individual ACC-Net genes commonly associates with increased mutational burden and reduced patient survival (Supplemental Fig. 4F). In additional, patients with a combined high expression of all ACC-Net genes showed significantly reduced survival (Supplemental Fig. 4G), comparable to that found in the TCGA dataset, and showed increased association with point mutations, insertions and deletions (Supplemental Fig. 4H).

As an experimental validation of ACC-Net genes playing a role in the DNA damage response, we conducted apoptosis assays in response to DNA damage along with siRNA mediated depletion of two ACC-Net target genes, KIF4A and MKI67. B*J*-5ta fibroblasts were treated with 25μM etoposide, a common ACC chemotherapeutic, after knockdown of our targets alongside a negative control scramble siRNA and to a positive control KU70 siRNA (Fig. 6H). Both KIF4A and MKI67 knockdown resulted in significantly increased apoptosis in response to etoposide treatment over the control siRNA (Fig. 6I). Interestingly, KIF4A depletion showed an increase in apoptosis similar to that of KU70 depletion, whereas MKI67 depletion resulted in an even greater increase than this, however these differences may be due to depletion efficiency (Fig. 6H). In addition, we repeated these experiments using 2μM cisplatin instead of etoposide as this is also a common ACC chemotherapeutic. This experiment gave very similar results to the etoposide treatment, with all siRNA showing significantly increased apoptosis compared to control siRNA and MKI67 siRNA showing an even greater increase than KIF4A or KU70 siRNA (Fig. 6I).

These results demonstrate that in ACC, this network of genes coordinates processes that lead to an increase in genomic mutations being maintained in the cancer genomes, which leads to progression of the cancer and, ultimately, an extraordinary decrease in patient survival. In addition, the corroboration of results for canonical DNA repair gene with those for the ACC-Net genes further supports that the ACC-Net genes operate via a DNA repair based mechanism.

## 3 Discussion

Here we showed the various features of Damage-Net, a program we built for meta-analysis and DNA repair investigation, along with further analysis that highlighted its effectiveness at identifying novel DNA repair genes. In addition, we found that there is an inherent aspect of irreproducibility in many datasets. The reason for this could simply be the large variation between experiments in different labs, as even results using very similar methodologies commonly give significantly different results. However, some publications have shown that even between biological replicates there is significant variability. For example, a recent publication conducted 3 replicates of affinity-purification mass-spec (AP-MS) and BioID of the Ku70 interactome and found a surprising lack of overlap between the AP-MS replicates [20]. Their BioID results were significantly more reproducible and identified a far greater number of significant hits suggesting that the variation in the AP-MS results may be method specific. This highlights the necessity of data integration for the selection of significant hits, as comparing your own datasets to comparable publishes datasets can filter out a majority of the irreproducible noise. Damage-Net provides a simple way to conduct such a comparison as well as other analytical techniques to aid the user’s projects.

To delve deeper into our database and to further assess the capabilities of Damage-Net, we integrated every Damage-Net dataset to identify a high confidence group of potential DNA repair genes which we termed DNet-genes. We found these genes were mainly associated with chromatin organisation and RNA-related processes, indicating that they may be required for chromatin remodelling in response to DNA repair, facilitating access to damage sites for repair factors. Expression of these genes was associated with increased tumour mutational burden and reduced cancer survival. These features were found to also be associated with canonical DNA repair gene expression and that in these features DNet-genes were very comparable to canonical repair genes. Given the expanding research implicating RNA processes and chromatin organisation genes in DNA repair, we hypothesise that these genes represent a large group of novel DNA repair genes that are either integral to canonical repair processes, or form distinct sub-processes that function alongside canonical repair processes. In addition, association with high mutational burden and poor cancer prognosis, indicates expression of these facilitates mutational burden via the repair of high levels of DNA damage. These mutations then confer an evolutionary advantage to the cancer cells, advancing their progression and providing the genetic heterogeneity necessary to adapt to environmental challenges, such as therapeutics, thus reducing patient survival.

Further investigation concluded that a majority of DNet-genes responsible for these changes do so primarily in adrenocortical carcinoma (ACC). We used hierarchical clustering to identify a co-expressing group of these ACC related genes and subsequently identified a network of genes that co-express with this entire group in ACC, termed ACC-Net genes. These genes all strongly co-express and contain cell-cycle and chromatin regulators as well as over 25 % being DNA repair factors. Every ACC-Net gene was associated with increased mutational burden and reduced survival in ACC and overall the expression of these genes had a greater effect on these features than canonical repair genes. Survival analysis found that overall expression of all these genes has a strong effect in ACC as high expression reduced 5-year survival from ∼80 % to ∼30 %, while increasing the average mutational burden by almost 2-fold and caused patients to be far more likely to have late-stage, metastatic ACC. Interestingly, multiple reports have implicated DNA repair and ACC-Net gene groups in ACC metastasis and prognosis [[60], [61], [62], [63]]. Metastatic ACC was previously shown to have substantially higher mutation rates than primary ACC and a hypermutation phenotype was identified to be associate with mutations in the DNA repair genes ATM, MSH3 and MSH6 [61]. Another study also identified mismatch repair genes, particularly MSH6, to be associated with high mutation frequency in ACC [60]. In addition, a smaller investigation found alterations in DNA repair genes, including ATM and BRCA2, to be associated with increased mutations and poor prognosis [63]. All these studies highlighted DNA repair, chromatin remodelling and cell cycle genes to be important markers for ACC prognosis and key to ACC metastasis, with some also suggesting the use of DNA repair inhibitors as a novel therapeutic strategy [60,62,63]. These studies further support our identification of the ACC-Net gene network as key prognostic markers and potential therapeutic targets.

We therefore hypothesise a mechanism by which these genes are required for DNA repair, and their elevated expression leads to increased mutation frequency at repair sites, overcoming the normally toxic levels of DNA damage to prevent apoptosis, but increasing mutational load. These mutations provide a large source of genetic variation, increasing the rate of evolution of the cancer, advancing its progression, and providing a greater ability for the cancer cells to adapt to and overcome therapeutics. All this contributes to the remarkable decrease in survival observed with high expression of these genes.

ACC is a relatively rare cancer but has quite poor prognosis for reasons that are not yet fully understood. Treatment options are limited and inadequate, leading to all treatments of unresectable and metastatic ACC to be solely palliative [64]. Prognosis has also been shown to be tightly linked to tumour grade, with 5-year survival of stage I patients being ∼82 % while for stage 4 patients it is as low as 6–13 % [60,[65], [66], [67]]. Some studies have found individual genes to be significant drivers of ACC, such as β-catenin and insulin-like growth factor 2 [68,69], however these individual genes often only apply to very few cases and offer limited mechanistic understanding and therapeutic possibilities. More recently, genomic investigations identified a number of genes associated with cell-cycle regulation and DNA replication repair as ACC prognostic markers [60,70] which were the first indications of dysregulation of these processes in ACC. Our findings here are therefore a significant step forward in our understanding of ACC pathogenesis. The original identification of these genes through Damage-Net and their correlations with mutational burden links their role directly to DNA repair, giving us the foundation for the likely mechanism by which they act, and the tumour stage and survival analysis presents a remarkable clinical result of the expression of these genes. Expression of these genes is therefore not only an effective prognostic marker but could also represent a large source of potential therapeutic targets, since their inhibition would theoretically reduce the cancers ability to progress and would improve patient survival. The large network of genes identified here gives multiple avenues for targeted therapeutics increasing the chance of success and allowing for combination therapy to reduce side-effects [[71], [72], [73]]. In addition, therapeutic targeting of these genes would likely be very effective as a combination therapy with traditional genotoxic therapeutics, such as cisplatin or radiotherapy, potentially increasing their efficacy at lower doses and thus reduced side-effects.

## 4 Conclusion

Damage-Net is a powerful tool in the investigation of DNA damage that greatly enhances users’ analysis of their large-scale results and also increases their ability to distinguish novel DNA repair factors from previously published datasets. With Damage-Net, we identified a network of novel DNA repair genes that are collectively responsible for a substantial decrease in adrenocortical carcinoma survival via facilitating increased mutational burden and therefore advanced cancer progression and treatment resistance. These represent a promising group of potential targets for therapeutics that could greatly enhance the efficacy of DNA damaging therapies, improving the currently dismal prognosis of ACC.

## 5 Materials and methods

### 5.1 Damage-Net access and installation

Damage-Net is available to download for all operating systems at www.damage-net.co.uk under downloads. This site also has information regarding installation and use and has a page for contacting us regarding problems, suggestions and questions.

The installers and source code are also available at https://sourceforge.net/projects/damage-net and the source is additionally available at https://github.com/aldob/Damage-Net

### 5.2 Damage-Net design

Damage-Net was written using Python 3.6.5. Multiple packages were used to create the variety of features such as SQL for writing the database and tkinter for writing the graphical interface. The following is the full list of all the packages used:

- lifelines v0.24.3
- matplotlib v3.1.3
- numpy v1.18.1
- pandas v0.25.3
- pillow v6.2.0
- seaborn v0.9.0
- sqlite v3.31.1
- tk v8.6.8
- tktreectrl v2.4.1
- venn v0.1.3

The scripts were compiled into an executable file using pyinstaller v3.5. The Windows installer was created using Inno Setup v6.0.3 and the Linux and MacOS installers were created using makeself v2.4.2.

### 5.3 Gene ontology enrichment analysis

Gene ontology enrichment was completed using PANTHER [74,75]. Term extraction was done by taking the lowest branch of each enrichment that had greater than 10 proteins identified and a p-value below 0.01. This was done to provide an informative and accurate balance between term specificity and statistical confidence.

The DNA repair gene ontology group was obtained from the MGI database [76].

### 5.4 Cancer mutation analysis

TCGA mutation and gene expression data were queried and downloaded using the TCGAbiolinks R package v2.10.5 [77]. The files obtained were of harmonised mutation data analysed using the MuTect2 pipeline in MAF file format. Gene expression data were downloaded in FPKM-UQ file format and combined per-patient with the mutation rates to create a database containing transcriptome wide gene expression and mutation frequencies per patient.

Tumour mutational burden was calculated as the total number of identified genomic mutations for a sample, i.e. point mutations + insertions + deletions. Mutation associations were calculated by dividing patients into two groups of high and low expression for a gene of interest, calculating the mean mutation frequency for each group and then calculating the log2 fold change of the high/low expression groups’ mutational frequencies.

### 5.5 Cancer survival analysis

TCGA survival data and cancer stage metadata were queried and downloaded using the TCGAbiolinks R package v2.10.5 [77]. Cox proportional hazards statistical analysis was used to calculate survival coefficients and p-values via the survival R package v3.1.8. Survival hazard ratios were generated by comparing groups of patients with either high or low expression of a gene of interest. This was then log2 transformed to make the data easier to understand by creating a value where positive values denote increased survival and negative values denote decreased survival.

Survival curves were generated using the survminer R package v0.4.6.

### 5.6 Network analysis

Protein interaction networks were modelled in Cytoscape v3.7.1 [78] using interaction strengths calculated using the STRING database [79]. Groups were designated based on the gene ontology biological process enrichment of the genes.

### 5.7 Co-expression analysis

Expression correlation was calculated between the expression of every gene being queried using the Pearson correlation model and the subsequent matrix was clustered via hierarchical clustering using the complete linkage model. The same matrix was also used for principal component analysis. The following base R functions were used for this analysis: cor.test, dist, hclust and prcomp. In addition, heatmap.2 from gplots v3.0.1.2 was used for heatmap plotting.

Strong correlation partners were extracted from transcriptome-wide correlation analysis by filtering the matrix for all genes that have a correlation value of > = 0.5 with over half of the target genes.

### 5.8 Cell-culture and transfection

BJ-5ta fibroblast cells were obtained from the ATCC and cultured in Dulbecco Modified Eagle′s Medium (DMEM, GibCo) fortified with 10 % Fetal bovine serum and 2 mM l-glutamine.

All siRNA transfections were completed using 20 nM siRNA with Dharmafect according to manufacturer instructions. The non-targeting control siRNA was obtained from Dharmacon (D-001810–03) and custom designed siRNA were obtained for KIF4A (UUAGAUGAUUAAGUUCAGC) [80], MKI67 (CGUCGUGUCUCAAGAUCUA) [81] and KU70 (GGAAGAGATAGTTTGATTT) [82] and ordered from Sigma.

### 5.9 Western blot

Protein samples were harvested by scraping cells in 1.2X sample preparation buffer (60 mM Tris pH 6.8, 12 % glycerol, 2.4 % SDS, 0.012 % bromophenol blue, 6% β-mercaptoethanol), sonicated in a Diagenode Bioruptor for 5 min on medium to shear the DNA and heated at 95C for 5 min. Samples were separated on polyacrylamide gels and transferred onto 0.45μM nitrocellulose membranes. The membranes were blocked in 5% BSA in TBST (0.1 % Tween-20) for 1 h at room temperature before probing overnight at 4C while rolling with the following antibodies and adilutions in 5% BSA TBST: MKI67 (Proteintech, 27309-1-AP, 1:1000), KIF4A (Santa Cruz, sc-365144, 1:500), KU70 (Santa Cruz, sc-5309, 1:1000), GAPDH (Santa Cruz, sc-32233, 1:10,000), gamma-H2AX (Sigma-Aldrich, 05-636, 1:1000). Membranes were then washed 3 times at room temperature in TBST for 10 min with rotation before probing with IR-Dye-labelled secondary antibodies (Li-COR Biosciences) at 1:10,000 in 5% BSA TBST for 1 h at room temperature with rotation. Membranes were again washed three times in TBST as before then scanned with a Li-COR Odyssey. Images were analyses using the Image Studio software.

### 5.10 Annexin-V apoptosis assay

BJ-5ta cells were seeded on six well plates at a density of 25,000 cells per well and incubated for 24 h. Cells were then transfected and incubated for 48 h in transfection media before this was replaced with treatment media containing either 25μM etoposide (Merck, E2600000) or an equivalent amount of DMSO, or in the case of cisplatin treatment media containing 2μM cisplatin or an equivalent amount of water. Cells were then incubated for a further 24 h and then cells were harvested with trypsin along with their media and PBS wash, pelleted at 300 g for 5 min, washed once in PBS and re-pelleted. Cells were then resuspended in 500 μL Annexin V binding buffer (10 mM HEPES pH 7.4, 140 mM NaCl, 2.5 mM CaCl) supplemented with 1 u L Annexin V-FITC (ab14082) and 5 u L 7-AAD (BioLegend, 420403) and incubated for 10 min in the dark before scanning on an Attune NxT flow cytometer.

### 5.11 R code

R version 3.5.0 was used for all analysis. All code used in data analysis and plot generation is available at https://github.com/aldob/Damage-Net/tree/master/publication.

## Funding source

The authors are funded by 10.13039/501100000289Cancer Research UK (core funding to the CRUK Beatson Institute A17196).

## Ethical approval and consent to participate

No work conducted for this manuscript involved human or animal participants and does not require ethical approval/consent.

## Consent to publish

All authors have read and approved the manuscript.

## Data availability

All data presented in this manuscript are open source and have been previously published, we have cited these publications appropriately.

## Author’s contributions

ASB built Damage-Net, conducted all data acquisition and analysis and drafted the manuscript, MB edited the manuscript.

## Author statement

Aldo S. Bader: Conceptualization, Methodology, Software, Validation, Formal analysis, Investigation, Data curation, Writing – Original draft preparation, Visualisation, Project administration. Martin Bushell: Writing – review & editing, Supervision, Funding acquisition.

## Declaration of Competing Interest

The authors report no declarations of interest.

## References

[1] Chatterjee N., Walker G.C. (2017). Mechanisms of DNA damage, repair, and mutagenesis. Environ. Mol. Mutagen..

[2] Jackson S.P., Bartek J. (2009). The DNA-damage response in human biology and disease. Nature.

[3] Wallace S.S. (2014). Base excision repair: a critical player in many games. DNA Repair.

[4] Schärer O.D. (2013). Nucleotide excision repair in eukaryotes. Cold Spring Harb. Perspect. Biol..

[5] Caldecott K.W. (2007). Mammalian single-strand break repair: mechanisms and links with chromatin. DNA Repair.

[6] Wright W.D., Shah S.S., Heyer W.D. (2018). Homologous recombination and the repair of DNA double-strand breaks. J. Biol. Chem..

[7] Hakem R. (2008). DNA-damage repair; the good, the bad, and the ugly. EMBO J..

[8] Torgovnick A., Schumacher B. (2015). DNA repair mechanisms in cancer development and therapy. Front. Genet..

[9] Loeb L.A., Loeb K.R., Anderson J.P. (2003). Multiple mutations and cancer. Proc. Natl. Acad. Sci. U. S. A..

[10] Veltman J.A., Brunner H.G. (2012). De novo mutations in human genetic disease. Nat. Rev. Genet..

[11] Claus E.B., Schildkraut J., Iversen E.S., Berry D., Parmigiani G. (1998). Effect of BRCA1 and BRCA2 on the association between breast cancer risk and family history. J. Natl. Cancer Inst..

[12] Romero-Laorden N., Castro E. (2017). Inherited mutations in DNA repair genes and cancer risk. Curr. Probl. Cancer.

[13] Chou D.M., Adamson B., Dephoure N.E., Tan X., Nottke A.C., Hurov K.E. (2010). A chromatin localization screen reveals poly (ADP ribose)-regulated recruitment of the repressive polycomb and NuRD complexes to sites of DNA damage. Proc. Natl. Acad. Sci. U. S. A..

[14] Maréchal A., Li J.M., Ji X.Y., Wu C.S., Yazinski S.A., Nguyen H.D. (2014). PRP19 transforms into a sensor of RPA-ssDNA after DNA damage and drives ATR activation via a ubiquitin-mediated circuitry. Mol. Cell.

[15] Xing M., Yang M., Huo W., Feng F., Wei L., Jiang W. (2015). Interactome analysis identifies a new paralogue of XRCC4 in non-homologous end joining DNA repair pathway. Nat. Commun..

[16] Chen Z., Tran M., Tang M., Wang W., Gong Z., Chen J. (2016). Proteomic analysis reveals a novel Mutator S (MutS) partner involved in mismatch repair pathway. Mol. Cell Proteomics.

[17] Beli P., Lukashchuk N., Wagner S., Weinert B., Olsen J., Baskcomb L. (2012). Proteomic investigations reveal a role for RNA processing factor THRAP3 in the DNA damage response. Mol. Cell.

[18] Jungmichel S., Rosenthal F., Altmeyer M., Lukas J., Hottiger M., Nielsen M. (2013). Proteome-wide identification of poly(ADP-ribosyl)ation targets in different genotoxic stress responses. Mol. Cell.

[19] Gupta R., Somyajit K., Narita T., Maskey E., Stanlie A., Kremer M. (2018). DNA repair network analysis reveals shieldin as a key regulator of NHEJ and PARP inhibitor sensitivity. Cell.

[20] Abbasi S., Schild-Poulter C. (2019). Mapping the Ku interactome using proximity-dependent biotin identification in human cells. J. Proteome Res..

[21] Adamson B., Smogorzewska A., Sigoillot F.D., King R.W., Elledge S.J. (2012). A genome-wide homologous recombination screen identifies the RNA-binding protein RBMX as a component of the DNA-damage response. Nat. Cell Biol..

[22] Antoniali G., Serra F., Lirussi L., Tanaka M., D’Ambrosio C., Zhang S. (2017). Mammalian APE1 controls miRNA processing and its interactome is linked to cancer RNA metabolism. Nat. Commun..

[23] Bennetzen M.V., Larsen D.H., Bunkenborg J., Bartek J., Lukas J., Andersen J.S. (2010). Site-specific phosphorylation dynamics of the nuclear proteome during the DNA damage response. Mol. Cell Proteomics.

[24] Bergink S., Theil A.F., Toussaint W., De Cuyper I.M., Kulu D.I., Clapes T. (2013). Erythropoietic defect associated with reduced cell proliferation in mice lacking the 26S proteasome shuttling factor Rad23b. Mol. Cell. Biol..

[25] Boeing S., Williamson L., Encheva V., Gori I., Saunders R.E., Instrell R. (2016). Multiomic analysis of the UV-induced DNA damage response. Cell Rep..

[26] Findlay S., Heath J., Luo V.M., Malina A., Morin T., Coulombe Y. (2018). SHLD2/FAM35A co-operates with REV7 to coordinate DNA double-strand break repair pathway choice. EMBO J..

[27] Elia A.H., Boardman A., Wang D., Huttlin E., Everley R., Dephoure N. (2015). Quantitative proteomic atlas of ubiquitination and acetylation in the DNA damage response. Mol. Cell.

[28] Herr P., Lundin C., Evers B., Ebner D., Bauerschmidt C., Kingham G. (2015). A genome-wide IR-induced RAD51 foci RNAi screen identifies CDC73 involved in chromatin remodeling for DNA repair. Cell Discov..

[29] Hill S.J., Rolland T., Adelmant G., Xia X., Owen M.S., Dricot A. (2014). Systematic screening reveals a role for BRCA1 in the response to transcription-associated DNA damage. Genes Dev..

[30] Izhar L., Adamson B., Ciccia A., Lewis J., Pontano-Vaites L., Leng Y. (2015). A systematic analysis of factors localized to damaged chromatin reveals PARP-dependent recruitment of transcription factors. Cell Rep..

[31] López-Saavedra A., Gómez-Cabello D., Domínguez-Sánchez M.S., Mejías-Navarro F., Fernández-Ávila M.J., Dinant C. (2016). A genome-wide screening uncovers the role of CCAR2 as an antagonist of DNA end resection. Nat. Commun..

[32] Matsuoka S., Ballif B.A., Smogorzewska A., McDonald E.R., Hurov K.E., Luo J. (2007). ATM and ATR substrate analysis reveals extensive protein networks responsive to DNA damage. Science.

[33] Mengwasser K.E., Adeyemi R.O., Leng Y., Choi M.Y., Clairmont C., D’Andrea A.D. (2019). Genetic screens reveal FEN1 and APEX2 as BRCA2 synthetic lethal targets. Mol. Cell.

[34] Olivieri M., Cho T., Álvarez-Quilón A., Li K., Schellenberg M., Zimmermann M. (2019).

[35] Povlsen L.K., Beli P., Wagner S.A., Poulsen S.L., Sylvestersen K.B., Poulsen J.W. (2012). Systems-wide analysis of ubiquitylation dynamics reveals a key role for PAF15 ubiquitylation in DNA-damage bypass. Nat. Cell Biol..

[36] Srivastava M., Chen Z., Zhang H., Tang M., Wang C., Jung S.Y. (2018). Replisome dynamics and their functional relevance upon DNA damage through the PCNA interactome. Cell Rep..

[37] Zimmermann M., Murina O., Reijns M.A.M., Agathanggelou A., Challis R., Tarnauskaitė Ž. (2018). CRISPR screens identify genomic ribonucleotides as a source of PARP-trapping lesions. Nature.

[38] Klont F., Bras L., Wolters J.C., Ongay S., Bischoff R., Halmos G.B. (2018). Assessment of sample preparation bias in mass spectrometry-based proteomics. Anal. Chem..

[39] Mellacheruvu D., Wright Z., Couzens A.L., Lambert J., St-Denis N., Li T. (2013). The CRAPome: a contaminant repository for affinity purification–mass spectrometry data. Nat. Methods.

[40] Piehowski P.D., Petyuk V.A., Orton D.J., Xie F., Moore R.J., Ramirez-Restrepo M. (2013). Sources of technical variability in quantitative LC-MS proteomics: human brain tissue sample analysis. J. Proteome Res..

[41] Sorace J.M., Zhan M. (2003). A data review and re-assessment of ovarian cancer serum proteomic profiling. BMC Bioinf..

[42] Wegler C., Gaugaz F.Z., Andersson T.B., Wiśniewski J.R., Busch D., Gröer C. (2017). Variability in mass spectrometry-based quantification of clinically relevant drug transporters and drug metabolizing enzymes. Mol. Pharm..

[43] Feng X., Luo Z., Jiang S., Li F., Han X., Hu Y. (2013). The telomere-associated homeobox-containing protein TAH1/HMBOX1 participates in telomere maintenance in ALT cells. J. Cell. Sci..

[44] Zhou S., Xiao Y., Zhuang Y., Liu Y., Zhao H., Yang H. (2017). Knockdown of homeobox containing 1 increases the radiosensitivity of cervical cancer cells through telomere shortening. Oncol. Rep..

[45] Fang F., Xia N., Angulo B., Carey J., Cady Z., Durruthy-Durruthy J. (2018). A distinct isoform of ZNF207 controls self-renewal and pluripotency of human embryonic stem cells. Nat. Commun..

[46] Boros-Oláh B., Dobos N., Hornyák L., Szabó Z., Karányi Z., Halmos G. (2019). Drugging the R-loop interactome: RNA-DNA hybrid binding proteins as targets for cancer therapy. DNA Repair.

[47] Xia F., Taghian D.G., DeFrank J.S., Zeng Z., Willers H., Iliakis G. (2001). Deficiency of human BRCA2 leads to impaired homologous recombination but maintains normal nonhomologous end joining. Proc. Natl. Acad. Sci. U. S. A..

[48] Bau D., Fu Y., Chen S., Cheng T., Yu J., Wu P. (2004). Breast cancer risk and the DNA double-strand break end-joining capacity of nonhomologous end-joining genes are affected by *BRCA1*. Cancer Res..

[49] Germano G., Amirouchene-Angelozzi N., Rospo G., Bardelli A. (2018). The clinical impact of the genomic landscape of mismatch repair–deficient cancers. Cancer Discov..

[50] Parikh A.R., He Y., Hong T.S., Corcoran R.B., Clark J.W., Ryan D.P. (2019). Analysis of DNA damage response gene alterations and tumor mutational burden across 17,486 tubular gastrointestinal carcinomas: implications for therapy. Oncologist.

[51] Chakraborty A., Tapryal N., Venkova T., Horikoshi N., Pandita R.K., Sarker A.H. (2016). Classical non-homologous end-joining pathway utilizes nascent RNA for error-free double-strand break repair of transcribed genes. Nat. Commun..

[52] Bader A.S., Hawley B.R., Wilczynska A., Bushell M. (2020). The roles of RNA in DNA double-strand break repair. Br. J. Cancer.

[53] Domingo-Prim J., Endara-Coll M., Bonath F., Jimeno S., Prados-Carvajal R., Friedländer M.R. (2019). EXOSC10 is required for RPA assembly and controlled DNA end resection at DNA double-strand breaks. Nat. Commun..

[54] Liebelt F., Schimmel J., Verlaan – de Vries M., Klemann E., van Royen M.E., van der Weegen Y. (2019). Transcription-coupled nucleotide excision repair is coordinated by ubiquitin and SUMO in response to ultraviolet irradiation. Nucleic Acids Res..

[55] Liang F., Miller A.S., Tang C., Maranon D., Williamson E.A., Hromas R. (2020). The DNA-binding activity of USP1-associated factor 1 is required for efficient RAD51-mediated homologous DNA pairing and homology-directed DNA repair. J. Biol. Chem..

[56] Urulangodi M., Mohanty A. (2020). DNA damage response and repair pathway modulation by non-histone protein methylation: implications in neurodegeneration. J. Cell Commun. Signal..

[57] Whalen J.M., Dhingra N., Wei L., Zhao X., Freudenreich C.H. (2020). Relocation of collapsed forks to the nuclear pore complex depends on sumoylation of DNA repair proteins and permits Rad51 association. Cell Rep..

[58] Baggerly K.A., Morris J.S., Coombes K.R. (2004). Reproducibility of SELDI-TOF protein patterns in serum: comparing datasets from different experiments. Bioinformatics.

[59] Assié G., Letouzé E., Fassnacht M., Jouinot A., Luscap W., Barreau O. (2014). Integrated genomic characterization of adrenocortical carcinoma. Nat. Genet..

[60] Zheng S., Cherniack A.D., Dewal N., Moffitt R.A., Danilova L., Murray B.A. (2016). Comprehensive pan-genomic characterization of adrenocortical carcinoma. Cancer Cell.

[61] Gara S.K., Lack J., Zhang L., Harris E., Cam M., Kebebew E. (2018). Metastatic adrenocortical carcinoma displays higher mutation rate and tumor heterogeneity than primary tumors. Nat. Commun..

[62] Subramanian C., Cohen M.S. (2019). Over expression of DNA damage and cell cycle dependent proteins are associated with poor survival in patients with adrenocortical carcinoma. Surgery.

[63] Lavoie J., Csizmok V., Wang G., Williamson L., Marra M.A., Laskin J.J. (2019). Whole genome and transcriptome analysis (WGTA) of metastatic adrenocortical carcinoma (mACC). JCO.

[64] Else T., Kim A.C., Sabolch A., Raymond V.M., Kandathil A., Caoili E.M. (2014). Adrenocortical carcinoma. Endocr. Rev..

[65] Else T., Williams Ar, Sabolch A., Jolly S., Miller Bs, Hammer Gd. (2014). Adjuvant therapies and patient and tumor characteristics associated with survival of adult patients with adrenocortical carcinoma. J. Clin. Endocrinol. Metab..

[66] Fassnacht M., Johanssen S., Quinkler M., Bucsky P., Willenberg H.S., Beuschlein F. (2009). Limited prognostic value of the 2004 International Union Against Cancer staging classification for adrenocortical carcinoma. Cancer.

[67] Fassnacht M., Kroiss M., Allolio B. (2013). Update in adrenocortical carcinoma. J. Clin. Endocrinol. Metab..

[68] Tissier F., Cavard C., Groussin L., Perlemoine K., Fumey G., Hagnere A.M. (2005). Mutations of beta-catenin in adrenocortical tumors: activation of the Wnt signaling pathway is a frequent event in both benign and malignant adrenocortical tumors. Cancer Res..

[69] Giordano T.J., Thomas D.G., Kuick R., Lizyness M., Misek D.E., Smith A.L. (2003). Distinct transcriptional profiles of adrenocortical tumors uncovered by DNA microarray analysis. Am. J. Pathol..

[70] Zou Y., Jing L. (2019). Identification of key modules and prognostic markers in adrenocortical carcinoma by weighted gene co-expression network analysis. Oncol. Lett..

[71] Yardley Da. (2013). Drug resistance and the role of combination chemotherapy in improving patient outcomes. Int. J. Breast Cancer.

[72] Ashdown M.L., Robinson A.P., Yatomi-Clarke S., Ashdown M.L., Allison A., Abbott D. (2015). Chemotherapy for late-stage cancer patients: meta-analysis of complete response rates. F1000Research.

[73] Bayat Mokhtari R., Homayouni T.S., Baluch N., Morgatskaya E., Kumar S., Das B. (2017). Combination therapy in combating cancer. Oncotarget.

[74] Ashburner M., Ball C.A., Blake J.A., Botstein D., Butler H., Cherry J.M. (2000). Gene Ontology: tool for the unification of biology. Nat. Genet..

[75] The Gene Ontology Consortium (2018). The gene ontology resource: 20 years and still GOing strong. Nucleic Acids Res..

[76] Bult C.J., Blake J.A., Smith C.L., Kadin J.A., Richardson J.E., the Mouse Genome, Database Group (2018). Mouse Genome Database (MGD) 2019. Nucleic Acids Res..

[77] Colaprico A., Silva T.C., Olsen C., Garofano L., Cava C., Garolini D. (2015). TCGAbiolinks: an R/Bioconductor package for integrative analysis of TCGA data. Nucleic Acids Res..

[78] Shannon P., Markiel A., Ozier O., Baliga N.S., Wang J.T., Ramage D. (2003). Cytoscape: a software environment for integrated models of biomolecular interaction networks. Genome Res..

[79] Szklarczyk D., Gable A.L., Lyon D., Junge A., Wyder S., Huerta-Cepas J. (2018). STRING v11: protein–protein association networks with increased coverage, supporting functional discovery in genome-wide experimental datasets. Nucleic Acids Res..

[80] Hou G., Dong C., Dong Z., Liu G., Xu H., Chen L. (2017). Upregulate KIF4A enhances proliferation, invasion of hepatocellular carcinoma and indicates poor prognosis across human cancer types. Sci. Rep..

[81] Sun X., Bizhanova A., Matheson T.D., Yu J., Zhu L.J., Kaufman P.D. (2017). Ki-67 contributes to normal cell cycle progression and inactive X heterochromatin in p21 checkpoint-proficient human cells. Mol. Cell. Biol..

[82] Biehs R., Steinlage M., Barton O., Juhasz S., Kunzel J., Spies J. (2017). DNA double-strand break resection occurs during non-homologous end joining in G1 but is distinct from resection during homologous recombination. Mol. Cell.

