# Supplementary material for: Damage-Net: A program for DNA repair meta-analysis identifies a network of novel repair genes that facilitate cancer evolution
Source: DNA Repair (Amst). 2021 Sep;105:103158. doi: 10.1016/j.dnarep.2021.103158 (PMC8385418; doi:10.1016/j.dnarep.2021.103158)
Supplement: Supplementary file 3 [file mmc3.docx]

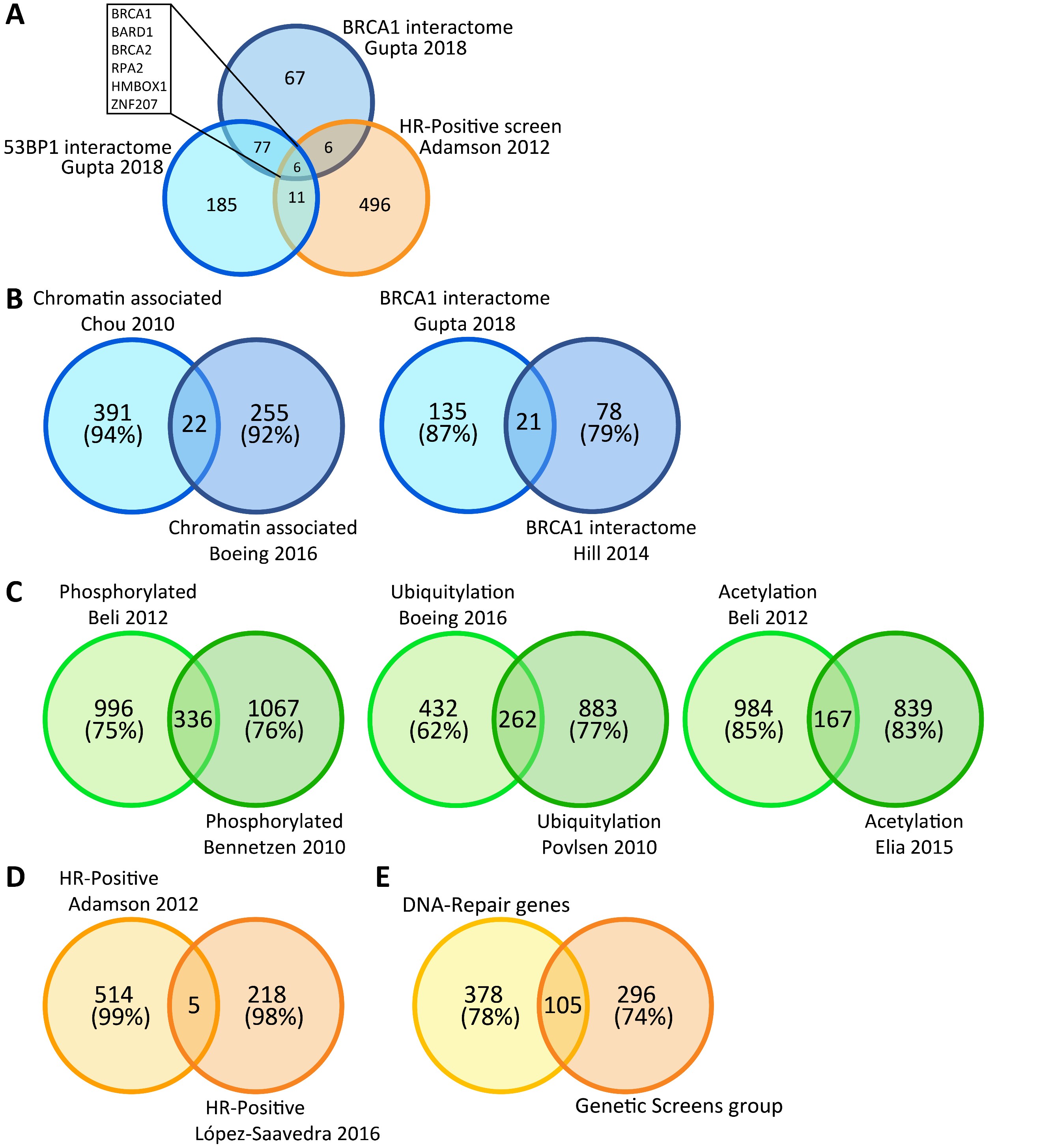


**Figure S1:** Comparisons of proteomic studies. (A) Three-way comparison of interactomes for 53BP1 and BRCA1 with a genetic screen for positive HR regulators. (B) Comparison of significant hits from 2 studies investigating chromatin associated proteins in response to damage (left) and 2 studies investigating BRCA1 interacting proteins. (C) Comparison of significant hits from 2 studies investigating proteins phosphorylated (left), ubiquitylated (middle) and acetylated (right) in response to damage. (D) Comparison of significant hits from 2 genetic screens using fluorescent reporters investigating homologous recombination (HR) promoting genes. (E) Comparison of the genes in the DNA repair gene ontology group compared with the significant hits from a collection of genetic screens defined in (Figure 2).


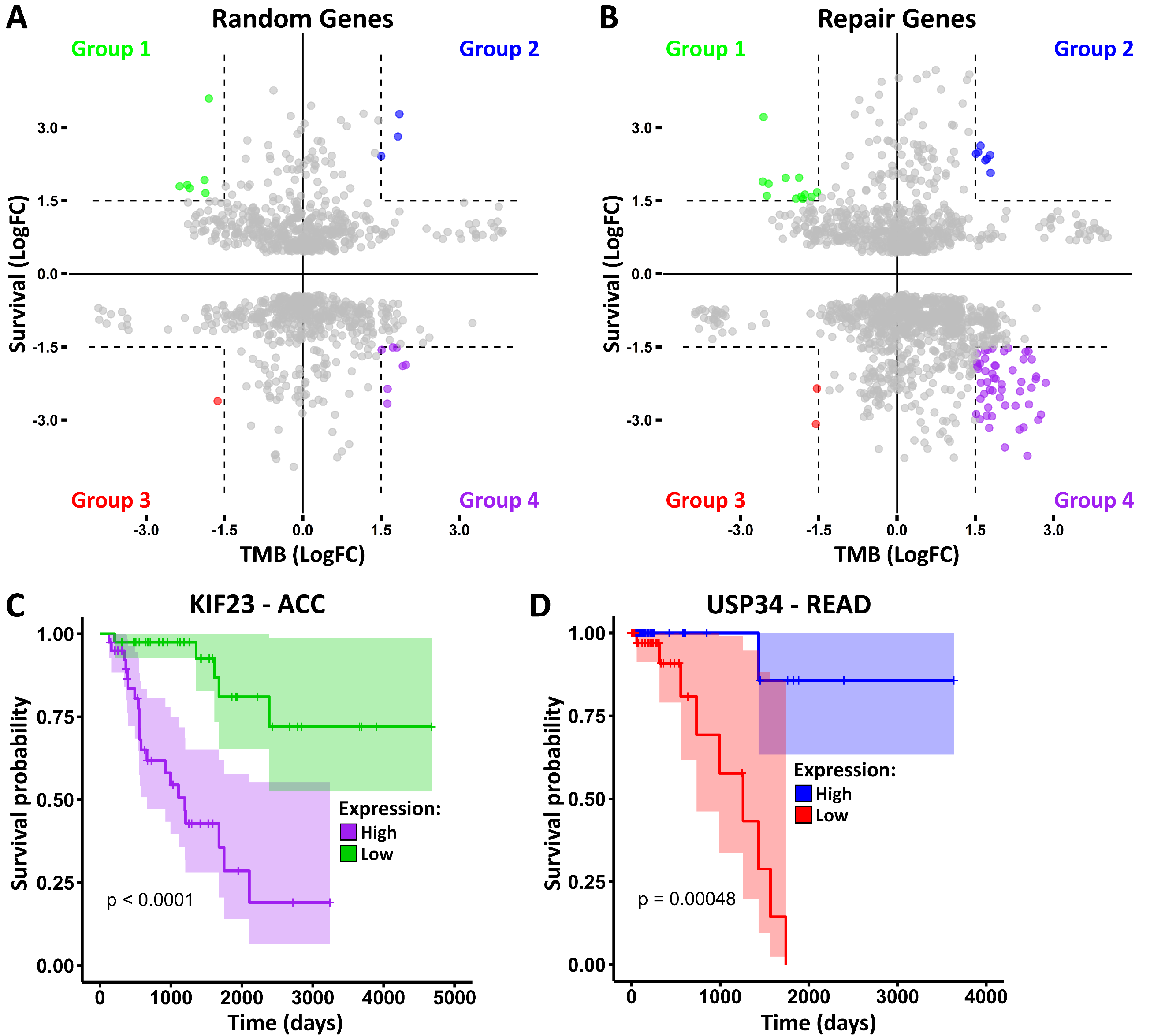


**Figure S2:** (A) Association with tumour mutational burden (TMB) vs association with survival of a group of randomly selected genes in all cancer sub-types with the 4 groups of cooperative change labelled groups 1-4. (B) Same as (A) but for canonical DNA repair genes. (C) Example survival curve for a group 4 DNet-gene, *KIF23* in adrenocortical carcinoma (ACC). (D) Example survival curve for a group 2 DNet-gene, *USP34* in rectal adenocarcinoma (READ).


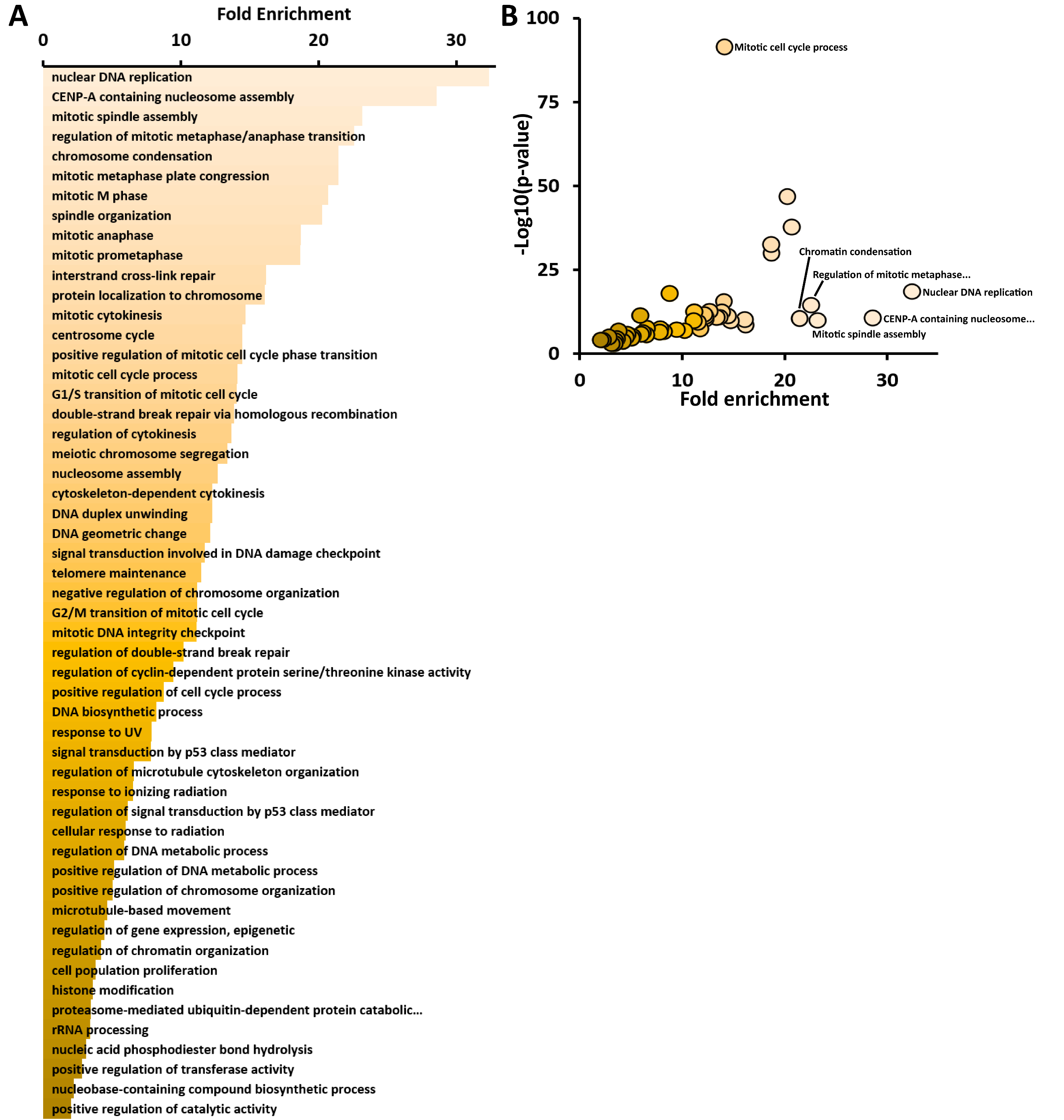


**Figure S3:** In depth gene ontology enrichment analysis of ACC-Net genes. (A) Full list of enriched terms for complete biological process enrichment, ranked by fold enrichment. (B) Scatter plot of -log10(p-value) vs fold enrichment of the GO terms from (A) with some strongly enriched terms labelled.


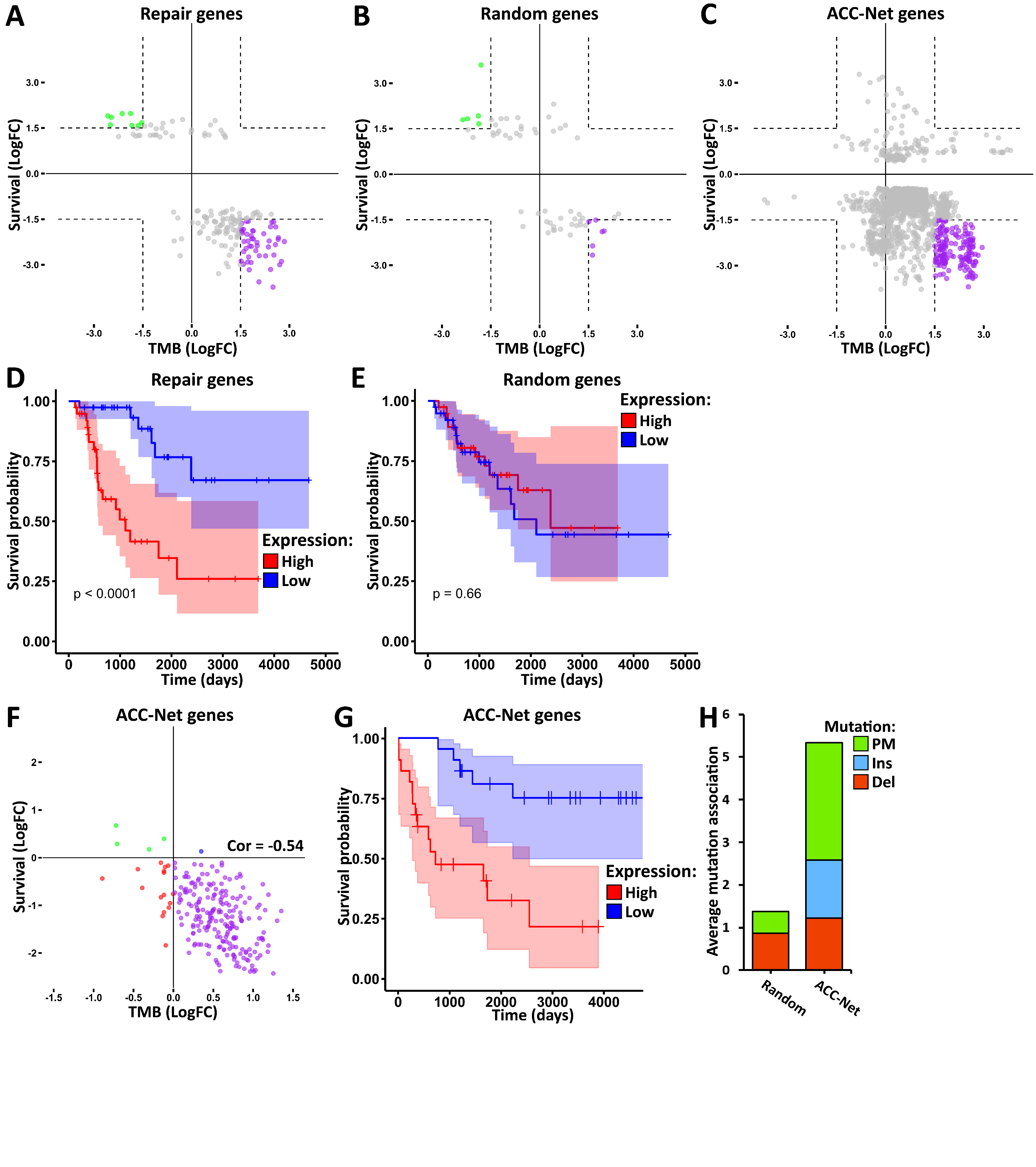


**Figure S4:** Effects of DNA repair vs random gene expression on adrenocortical carcinoma (ACC). (A) Association with tumour mutational burden (TMB) vs association with survival of all canonical DNA repair genes in ACC. (B) Same as (A) but for randomly selected genes. (C) Pan-cancer association with tumour mutational burden (TMB) vs association with survival of ACC-Net genes. (D) ACC survival curve of high vs low cumulative expression of all canonical DNA repair genes. (E) Same as (D) but for randomly selected genes. (E-F) Show data from an orthogonal dataset in G. Assié et al., 2014 (F) Association with tumour mutational burden (TMB) vs association with survival of all ACC-Net genes in ACC, Pearson correlation coefficient is marked. (G) ACC survival curve of high vs low cumulative expression of all ACC-Net genes. (H) Average mutation association of our three gene groups for point mutations, insertions and deletions separately.
